# Co-Production of Behavior Change Intervention Promoting an Anti-Inflammatory Diet for Frailty Prevention in Community-Dwelling Older Adults

**DOI:** 10.3390/nu18091420

**Published:** 2026-04-30

**Authors:** Weida Lyu, Momoka Masuda, Kozue Kubo, Hayato Isomoto, Yuki Tamii, Youko Nakamae, Yuka Okitsu, Asako Arai, Masako Ueno, Masahiro Akishita, Katsuya Iijima, Bo-Kyung Son

**Affiliations:** 1 Institute of Gerontology, The University of Tokyo, Tokyo 113-8656, Japan; 2 Department of Human Ecology, The University of Tokyo, Tokyo 113-8654, Japan; 3 Welfare for the Elderly in Toshima City, Tokyo 171-8422, Japan; 4 Tokyo Metropolitan Institute for Geriatrics and Gerontology, Tokyo 173-0015, Japan; 5 Institute for Future Initiatives, The University of Tokyo, Tokyo 113-0033, Japan; 6 Coproduction of Inclusion, Diversity and Equity (IncluDE) Center, The University of Tokyo, Tokyo 113-8654, Japan

**Keywords:** anti-inflammatory diet, co-production, DII, dietary fiber, frailty prevention, community

## Abstract

**Background/Objectives**: Chronic inflammation is a fundamental biological process underlying aging and frailty. We recently demonstrated that an anti-inflammatory diet, assessed using the Dietary Inflammatory Index (DII), was associated with serum high-sensitivity C-reactive protein levels and frailty incidence among community-dwelling older adults. The present study aimed to co-produce behavior change intervention promoting an anti-inflammatory diet by participatory action research with older adults. Particularly, increasing intake of dietary fiber was targeted as it represents a nutrient with the highest anti-inflammatory potential within the DII framework. **Methods**: Participants were community-dwelling older adults engaged in frailty checkup activity. Six co-production workshops were conducted between May 2022 and February 2023, integrating semi-structured group work and scientific evidence. Participant satisfaction was assessed after each session. Changes in dietary behavior were evaluated using DII score and dietary intake assessed by the Brief Self-Administered Diet History Questionnaire (BDHQ). **Results**: A cumulative total of 66 participants was involved (mean age, 73.7 ± 4.8 years; 80.0% women). When compared before and after co-production workshops, total DII scores and DII scores calculated by anti-inflammatory nutrients significantly decreased (*p* = 0.031 and *p* = 0.020, respectively). Dietary fiber intake also significantly increased following the workshop (*p* = 0.044). Among dietary fiber-rich food groups, mushroom consumption showed a particularly significant increase (*p* = 0.048). **Conclusions**: Co-production workshops integrating group work and scientific evidence were effective in promoting behavioral changes toward an anti-inflammatory diet among community-dwelling older adults. This developed intervention may represent a feasible and practical dietary strategy for frailty prevention in community settings.

## 1. Introduction

Frailty is a major geriatric syndrome characterized by decreased physiological reserve and increased vulnerability to stressors, resulting in adverse health outcomes such as disability, hospitalization, and mortality [1,2]. Chronic inflammation, commonly referred to as inflammaging, has been recognized as a fundamental biological mechanism underlying frailty and age-related diseases including sarcopenia [3,4,5].

Diet is one of the key modifiable determinants of chronic inflammation. The Dietary Inflammatory Index (DII) has been developed as a validated tool to assess the inflammatory potential of overall dietary intake [6]. Recently, we demonstrated that a more pro-inflammatory diet, indicated by higher DII scores, is associated with sarcopenia and frailty, as well as circulating high-sensitivity C-reactive protein (hsCRP) levels in community-dwelling older adults [7,8]. Furthermore, we observed that higher DII scores were primarily attributed to low intake of anti-inflammatory food components rather than high intake of pro-inflammatory foods. Recent evidence also supports that plant-based dietary patterns exert anti-inflammatory effects through lowering DII scores and further, individuals following vegan diets exhibit significantly lower circulating inflammatory biomarkers, including hsCRP and IL-6, compared with omnivores [9,10]. These findings highlight the importance of promoting anti-inflammatory dietary pattern among community-dwelling older adults as a potential strategy for frailty prevention.

Within the DII framework, anti-inflammatory dietary patterns are characterized by higher intakes of fiber-rich foods, which have strong anti-inflammatory potential and confer benefits to gut health [11,12,13]. Despite growing evidence linking anti-inflammatory diets to healthy aging, few community-based dietary interventions specially targeting anti-inflammatory dietary behaviors for frailty prevention have been developed.

For older adults, to ensure feasibility and effectiveness, interventions must consider individual dietary habits and preferences. Recently, the Medical Research Council has highlighted the importance of combining evidence, theory, and stakeholder involvement throughout the intervention development process [14]. However, there remains a lack of interventions promoting anti-inflammatory dietary behaviors in older adults that integrate participatory action research with theory-driven behavior change frameworks and scientific evidence.

In the present study, we aimed to co-produce behavior change intervention promoting an anti-inflammatory diet among community-dwelling older adults, guided by the Behavioral Change Wheel (BCW) framework, a comprehensive and theory-based framework for designing behavior change interventions through capability, opportunity, motivation, and the use of intervention function [15,16].

The specific objectives were:

(1) To conduct co-production group works with older adults, integrating scientific evidence to design an intervention targeting anti-inflammatory dietary behaviors;
(2) To evaluate the effectiveness of the developed intervention in improving dietary behavior using DII scores and food intake;
(3) To develop practical intervention materials to facilitate behavior change toward an anti-inflammatory diet in community-dwelling older adults.

## 2. Materials and Methods

### 2.1. Participants

Participants were recruited from voluntary community-based supporters involved in frailty checkup activities. A cumulative total of 66 attendances was recorded in the co-production workshops conducted between May 2022 and February 2023. The actual number of unique participants was 25, as some individuals attended multiple sessions (Table S1). The study protocol was reviewed and approved by the Institutional Review Committee of the University of Tokyo (#22–40, 28 April 2022). All participants received a comprehensive explanation of the study objectives and written informed consent was obtained prior to participation.

### 2.2. Co-Production Workshops

The co-production workshops were developed based on BCW framework, which provided a systematic and theory-driven approach for designing behavior change interventions [17]. This framework is particularly suitable for community-based interventions, as it integrates behavioral theory, evidence, and stakeholder involvement to enhance feasibility and implementation.

A total of six workshop sessions was conducted once every 2 months for 1 year, each lasting approximately 90 min. Each session consisted of three structed components: (1) **Introduction and information sharing**: Essential information relevant to the current session and an overview of previous sessions were provided during the workshops’ first 10–15 min. (2) **Group-work activities**: Participants engaged in semi-structured group work consisting of one or two activities per session. Small subgroups comprising five to six older adults and one researcher collaboratively discussed dietary behaviors and ideation to promote anti-inflammatory dietary practice. (3) **Workshop evaluation and feedback**: At the end of each session, subgroups reconvened and presented their discussion to all participants.

Among the 15 participants with comparable dietary behavior change data, individuals attended an average of 4.0 ± 1.0 sessions out of six workshops, suggesting that a structured, multi-session educational approach—rather than the provision of simple information alone—may be important for encouraging behavior change. The intervals between sessions were designed to allow sufficient time for self-reflection and practical application in daily life in an older population.

Participants also completed an evaluation questionnaire assessing workshop satisfaction and stage of behavior change based on the Transtheoretical Model including precontemplation, contemplation, preparation, action, and maintenance stage [18].

### 2.3. Dietary Assessment

Dietary intake was assessed using the brief self-administered diet history questionnaire (BDHQ), previously validated [19,20]. The Dietary Inflammatory Index (DII) was calculated according to the method developed by Shivappa et al. [6]. Briefly, the DII score was derived using intake data for 25 dietary parameters obtained from the BDHQ, including alcohol, vitamin B12, vitamin B6, b-carotene, carbohydrate, cholesterol, total fat, fiber, folic acid, iron, magnesium, monounsaturated fatty acids, niacin, n-3 fatty acids, n-6 fatty acids, protein, polyunsaturated fatty acids, riboflavin, saturated fat, thiamine, vitamin A, vitamin C, vitamin E, zinc, and tea. The overall DII score reflects the inflammatory potential of the diet, with higher scores indicating more pro-inflammatory dietary patterns. In addition, pro-inflammatory and anti-inflammatory DII scores were calculated using positive (inflammation inducible 7 parameters) and negative (inflammation repressive 18 parameters) nutrients, respectively.

### 2.4. Other Assessments

Height and weight were reported to calculate body mass index (BMI). Mental health was assessed using the WHO-5 Well-Being Index, a short self-reported questionnaire consisting of five items, with total scores ranging from 0 to 25, where higher scores indicate better well-being. Daily life satisfaction, number of meals per day, and exercise habits were assessed using self-administered questionnaires.

### 2.5. Statistical Analysis

Before and after the workshops, DII scores, and nutrient and food intake were compared using the Wilcoxon signed-rank test for continuous variables. Effect sizes were also calculated to quantify the magnitude of the observed effect and to complement statistical significance indicated by *p*-values. Participant satisfaction and stage of behavior during the second and fifth workshop sessions were compared using chi-square test for categorical variables. All statistical analyses were performed using IBM SPSS statistics version 29 for Windows (IBM Japan, Tokyo, Japan). Statistical significance was defined as a *p*-value < 0.05.

## 3. Results

In the co-production workshop, a cumulative total of 66 attendances by community-dwelling older adults were recorded across multiple sessions. Due to repeated participation, the actual number of unique participants was 25. Table 1 summarizes the characteristics of these participants. The mean age was 73.7 years, and 80.0% were women. The majority of participants (93.7%) rated their self-perceived health as good or very good. The mean WHO-5 score was 17.5, indicating a generally favorable mental health status [21]. In addition, 80.0% of participants reported satisfaction with their daily lives, and 86.7% reported consuming three meals per day. Regarding exercise habits, 55.6% of participants engaged in regular physical activity, defined as exercising at least twice per week for ≥30 min per session for more than one year.

All participants in the co-production workshop were actively involved in community-based frailty checkup activities as frailty supporters. This role was strategically positioned to enable them to act as peer-advisors, facilitating the dissemination of anti-inflammatory dietary practices among other community-dwelling older adults participating in frailty checkup programs.

Table 2 presents the contents of the co-production workshops designed to promote an anti-inflammatory diet. The six sessions were structured to facilitate awareness, knowledge acquisition, and behavioral application:

(1) **Session 1** focused on increasing awareness of individual dietary habits through self-reflection and group discussion.
(2) **Session 2** aimed to enhance understanding of the relationship between diet, inflammation, and immune function across the life course.
(3) **Session 3** emphasized the role of dietary fiber in anti-inflammatory diets, highlighting gut health, gut microbiota, and immune response, particularly the function of fiber as a prebiotic.
(4) **Session 4** addressed frailty-preventive dietary strategies emphasizing the combined importance of adequate protein intake and fiber-rich foods.
(5) **Session 5** focused on identifying personalized dietary practices that were feasible and sustainable in participants’ daily lives.
(6) **Session 6** emphasized behavior change techniques for community-dwelling older adults, facilitating the integration of acquired knowledge and individual experiences into community settings.

**Figure 1:**
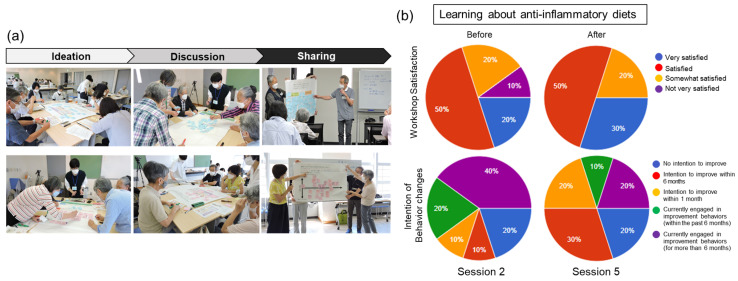
Group work activity (**a**) and pie charts for workshop satisfaction and intention of behavior changes (**b**).

Figure 1 illustrates the group work and board-based presentations (Figure 1a), as well as the results of the workshop satisfaction and behavior intention assessments (Figure 1b). Regarding workshop satisfaction, the proportion of participants reporting being “very satisfied” tended to increase following the session on anti-inflammatory diets (*p* = 0.057, *n* = 10). With respect to behavior intention, the proportion of participants intending to improve their dietary habits within 6 months and within 1 month increased; however, these changes were not statistically significant (*p* = 0.240), likely due to the small sample size (*n* = 10) and the allowance of multiple responses.

To examine changes in dietary behaviors following the workshops, we compared DII score and nutrient intakes before and after the intervention, as shown in Table 3. Before–after comparisons were available for 15 participants who engaged in the workshops, attending an average of 4.0 ± 1.0 sessions out of six sessions. The total DII score significantly decreased after the workshops (Z = −2.158, *p* = 0.031) with a large effect size (r = 0.557). A similar significant reduction was observed in the anti-inflammatory DII score (Z = −2.329, *p* = 0.020, effect size r = 0.601), whereas no significant change was observed in the pro-inflammatory DII score (*p* = 0.281).

Among nutrients, the intakes of total dietary fiber, which has the highest anti-inflammatory potential (DII score: −0.663), significantly increased following the workshops (Z = −1.817, *p* = 0.044, effect size r = 0.469). Intakes of other anti-inflammatory nutrients, including vitamin B6 (DII score: −0.365), vitamin C (DII score: −0.424), magnesium (Mg; DII score: −0.484), and folic acid (DII score: −0.190), also showed increasing trends after the intervention. Carbohydrate intake (DII score: 0.097) also significantly increased after the workshops.

Regarding changes in food group intakes, among dietary fiber-rich foods such as sea weeds, mushrooms, and legumes, mushroom intake significantly increased following the workshop (Z = −1.978, *p* = 0.048, effect size r = 0.511), as shown in Table 4. Intake of potatoes and tubers also tended to increase (*p* = 0.071).

## 4. Discussion

In the present study, we co-produced an intervention promoting an anti-inflammatory diet for frailty prevention with community-dwelling older adults, focusing on dietary fiber which is the highest anti-inflammatory potential nutrient within the DII framework. Through six co-production workshops integrating group work and scientific evidence, we observed behavior changes to anti-inflammatory dietary patterns, as demonstrated by a significant reduction in total DII scores and increased dietary fiber intake. Furthermore, among fiber-rich foods, mushroom intake significantly increased following the workshops, suggesting that co-production workshops incorporating practical strategies may facilitate the adoption of anti-inflammatory dietary patterns among community-dwelling older adults.

In the co-production workshop, participants were first provided with scientific evidence demonstrating a positive association between inflammatory dietary patterns and frailty risk [22]. Subsequently, we introduced findings from our recent longitudinal study showing that higher DII scores reflecting low intake of anti-inflammatory nutrients were associated with an increased frailty incidence over a 7-year follow-up period [8]. These findings likely enhanced participants’ interest in dietary fiber as a key anti-inflammatory component, particularly given its biological role as a prebiotic influencing gut microbiota and immune regulation [23,24,25]. Importantly, the acquisition of evidence-based knowledge during the workshop may have served as a critical step in increasing participants’ motivation, thereby facilitating the co-production of practical dietary interventions. Although frailty outcomes were not assessed in the present study, further investigations are warranted to validate the potential preventive effects of anti-inflammatory dietary behaviors on frailty incidence.

Co-production represents a valuable participatory research approach that actively involves target populations in the development of interventions [26]. By engaging older adults as shared decision-makers, co-production enhances the feasibility, acceptability, and appropriateness of interventions for the intended population [27,28,29,30]. In the present study, small group discussions provided a supportive environment that enabled participants to actively share experiences, exchange ideas, and engage in constructive dialog.

The iterative nature of the co-production process allowed older adults to observe how their opinions were actively shaping the intervention [30]. Consistent with previous studies reporting the benefits of co-production in dietary intervention development [31,32], our findings demonstrated high workshop satisfaction and favorable behavioral changes toward anti-inflammatory dietary practices.

Another well-received aspect of co-production was the social interactions the group setting provided. Participants explicitly referred to the value of shared experiences and peer support when identifying feasible dietary practices. In particular, the significant increase in mushroom intake observed in this study may reflect the practical dietary strategies discussed during Session 4, in which participants explored fiber-rich food options such as sea weeds, mushrooms, and legumes. These findings suggest that participatory group discussions may promote the translation of knowledge into actionable dietary behaviors. Furthermore, in our previous participatory action research involving older adults, co-production was associated with significant improvements in self-efficacy, particularly in the domain of social role and capability [33]. These findings collectively highlight the potential of co-production not only to improve dietary behaviors but also to enhance psychological factors that support sustainable health behavior change.

## 5. Limitations

This study has several limitations. First, the sample size was relatively small for evaluating dietary behavioral changes through the workshop; thus, larger-scale community-based interventions with sex- and gender-balanced participants are warranted. Second, no control group was included to assess the effectiveness of the co-production approach for behavioral change. Third, approximately 80% of participants were women, which may have influenced both the co-production process and its outcomes across sex and gender. In Japan, cooking is predominantly undertaken by women among older adults, and in community-based interactions, cooking skills may serve as a key mediator linking daily behaviors with social capital, thereby facilitating the maintenance of stronger interpersonal networks [34,35]. In this context, anti-inflammatory intervention might be effective not only for women participants, but also for men who live together. As another concern, alternative strategies are needed to engage older men, particularly those living alone, including creating opportunities and motivation for cooking-related activities. Fourth, participants were frailty supporters who already had relatively high awareness of frailty prevention and healthy dietary practices, and thus the generalizability to the broader older population remains unclear. However, frailty supporters were intentionally recruited in this co-production workshop to facilitate intervention development, as peer-led approaches among community-dwelling older adults have been shown to effectively promote sustained behavioral change [36,37,38,39]. As peer advisors, they may play a key role in disseminating and facilitating anti-inflammatory dietary practices within community-based frailty checkup activities. Nevertheless, future studies incorporating larger samples and appropriate control groups (e.g., dietitian-led interventions) are needed to validate the effectiveness of peer-led strategies.

## 6. Conclusions

In this study, we developed and implemented co-produced, community-based intervention aimed at promoting anti-inflammatory dietary behaviors focusing on dietary fiber among community-dwelling older adults. Co-production workshops were designed as an innovative approach integrating group work and scientific evidence. We confirmed that this participatory approach was associated with favorable changes in dietary patterns, including a significant reduction in dietary inflammatory potential and increased intake of dietary fiber-rich foods. These results suggest that co-production may represent a feasible and acceptable strategy for facilitating dietary behavior change in older populations. Importantly, this study highlights the potential of engaging older adults in intervention design to enhance motivation, practicality, and sustainability of health behaviors. The observed improvements in specific dietary components, such as increased mushroom consumption, further underscore the value of translating knowledge into actionable practices through interactive group processes.

However, given the methodological limitations, including the small sample size and gender imbalance, the present findings should be interpreted with caution. While the intervention was designed within a frailty prevention framework, future studies incorporating larger, more diverse populations, controlled study designs, and direct assessments of frailty indicators are warranted to establish causal effects and clinical relevance.

Despite these limitations, this study provides novel insights into the application of co-production methodologies for promoting anti-inflammatory dietary behaviors in aging populations. Our findings lay the groundwork for future research aimed at developing scalable, community-based strategies to reduce inflammation-related health risks and support healthy aging.

## Figures and Tables

**Table 1:** Characteristics of participants.

	Ave ± SD or %
Age (years, AVE ± SD)	73.7 ± 4.8
Women (%)	80.0 (%)
BMI (kg/m^2^)	22.5 ± 2.8
Subjective health (%, very good or good)	93.7%
Mental health ^1^ (points)	17.5 ± 5.2
Daily life satisfaction (%, satisfied or somewhat satisfied)	80.0%
3 meal-time a day (%, yes)	86.7%
Exercise habit (%, more than 2 times a week, 30 min a time, more than 1 year)	55.6%

AVE indicates the average, and SD indicates the standard deviation. ^1^ Mental health was assessed using the World Health Organization-five Well-Being Index (WHO-5), with total scores ranging from 0 to 25. Higher scores indicate better mental health.

**Table 2:** Overview of workshop contents for an anti-inflammatory diet (90 min session).

	Session 1	Session 2	Session 3	Session 4	Session 5	Session 6
Workshop Title	**Awareness of** **Dietary Habits**	**Understanding the Relationship Between Diet and Immune Function**	**Immunity, Gut Health, and Dietary Fiber**	**Effective Dietary Practices for Frailty Prevention**	**Identifying Personalized** **Dietary Practice**	**Developing Materials for Practical Intervention**
Workshop Topics	Distinguish between eating for health and eating based on preference	Understand dietary trajectories and triggers for eating behavior change	Understand anti-inflammatory diets, dietary fiber, and immune function	Apply evidence-based dietary strategies: combined intake of protein and dietary fiber	Identify sustainable, personalized anti-inflammatory dietary practices	Consolidate individual ideas and recipes
Group Work	**Self-reflection on food awareness and preferences** (1) Identification of the five most frequently consumed foods/dishes (2) Identification of foods consumed with awareness of frailty prevention (3) Identification of favorite foods among the five	**Triggers, actions, and** **behavioral change** Participants share at least 3 episodes where they decided to improve their eating habits	**Sharing practical know-how to boost immunity and their source**	**Practical dietary strategies for frailty prevention** (1) Trial fiber-rich foods (seaweed, mushrooms, and legumes) (2) Strategies for combined intakes of dietary fiber and protein (3) Dietary reflection from a gut microbiota perspective	**Sharing experience on** **incorporating fiber-rich foods into daily diets**	**Collection of** **academic knowledge, practical recipes, and** **nutritional expert advice**
		**What would you do?** **Advice simulation game** Persona-based advice simulation on dietary practices to enhance immunity (before learning about anti-inflammatory diets)	**Card sorting game** (1) Sorting food cards according to dietary fiber content (2) Selection of fiber-rich foods relevant to daily diets		**“My recommended advice” simulation game** (1) Consolidation of practical advice for anti-inflammatory diet in community settings (2) Practice in advising other older adults through role-play discussions (after acquiring knowledge about anti-inflammatory diet)	
Scientific Evidence			**Lecture**: Improving immunity through food and nutrition for frailty prevention: gut environment, and the role of dietary fiber	**Lecture**: Integrating anti-inflammatory diets and protein intake for frailty prevention		
Assessment	Food habits survey Feedback on group work	Feedback on group work	Feedback on group work	Feedback on group work	Feedback on group work	Food habits survey Feedback on group work

**Table 3:** DII score and nutrients intakes before and after dietary practice program.

	Before	After	*p*
BMI (kg/m^2^)	22.5 ± 2.8	22.8 ± 2.6	0.158
Total DII	0.40 ± 2.03	−0.18 ± 2.04	0.031 *
Pro-inflammatory DII	−0.14 ± 0.60	−0.03 ± 0.65	0.281
Anti-inflammatory DII	0.53 ± 2.53	−0.20 ± 2.65	0.020 *
Energy (Kcal/day)	1984.7 ± 626.0	2130.2 ± 631.7	0.125
Protein (g)	107.6 ± 57.1	110.5 ± 50.3	0.334
Fat (g)	73.1 ± 26.0	76.4 ± 23.4	0.363
Carbohydrate (g)	211.9 ± 63.1	238.4 ± 79.5	0.023 *
Cholesterol (mg)	665.4 ± 387.7	613.7 ± 268.3	0.955
SFA (g)	18.6 ± 6.1	19.4 ± 5.7	0.394
PUFA (g)	18.0 ± 6.9	18.8 ± 5.7	0.363
β-carotene (µg)	6603.1 ± 3419.9	6993.2 ± 2974.1	0.650
Retinol (µg)	757.6 ± 445.7	697.9 ± 338.9	0.570
Vit B1 (mg)	1.2 ± 0.5	1.3 ± 0.4	0.176
Vit B2 (mg)	2.1 ± 0.9	2.1 ± 0.7	0.712
Niacin(mg)	27.3 ± 14.9	29.4 ± 14.9	0.156
Vit B6 (mg)	2.0 ± 1.0	2.2 ± 0.9	0.073 ^#^
Vit B12 (µg)	19.2 ± 17.4	20.1 ± 18.3	0.691
Vit C (mg)	185.5 ± 83.8	209.9 ± 82.5	0.078 ^#^
Vit D (µg)	30.8 ± 34.2	33.2 ± 34.3	0.609
α-tocopherol (mg)	11.3 ± 4.3	11.8 ± 3.9	0.293
Total dietary fiber (g)	17.7 ± 7.2	19.7 ± 6.4	0.044 *
Mg (mg)	379.4 ± 191.3	407.0 ± 153.2	0.053 ^#^
Folic acid (μg)	540.4 ± 248.4	567.0 ± 167.1	0.061 ^#^
Alcohol (g)	4.9 ± 10.1	5.3 ± 9.3	0.499
w3 fatty acid (g)	4.5 ± 2.7	4.8 ± 3.1	0.394
w6 fatty acid (g)	13.4 ± 4.7	13.9 ± 3.3	0.307

Note: ^#^ *p* < 0.1, * *p* < 0.05. Data were available for 15 participants. DII: Dietary Inflammatory Index; SFA: saturated fatty acid; PUFA: polyunsaturated fatty acid.

**Table 4:** Food intake before and after dietary practice program.

	Before	After	*p*
Cereals and grains (g)	260.1 ± 118.8	274.4 ± 113.9	0.460
Potatoes and tubers (g)	75.9 ± 60.6	106.73 ± 90.7	0.071 ^#^
Legumes (g)	113.7 ± 80.3	124.3 ± 68.1	0.363
Green and yellow vegetables (g)	419.8 ± 181.8	424.1 ± 148.0	0.427
Mushrooms (g)	20.4 ± 10.3	27.1 ± 17.5	0.048 *
Sea weeds (g)	24.2 ± 19.0	28.5 ± 17.8	0.272
Fruits (g)	130.7 ± 84.1	159.1 ± 122.1	0.211
Fish and shellfish (g)	132.2 ± 129.9	170.7 ± 171.1	0.112
Meat (g)	114.9 ± 36.2	114.3 ± 35.5	0.865
Eggs (g)	71.2 ± 39.2	57.6 ± 19.1	0.509
Milk and dairy products (g)	198.0 ± 96.3	203.4 ± 153.4	0.826
Fats and oils (g)	12.3 ± 4.4	11.4 ± 3.2	0.570

Note: ^#^ *p* < 0.1, * *p* < 0.05. Data were available for 15 participants.

## Data Availability

The data that support the findings of this study are available on request from the corresponding author. The data are not publicly available due to privacy or ethical restrictions.

## Supplementary Materials

The following supporting information can be downloaded at: https://www.mdpi.com/article/10.3390/nu18091420/s1, Table S1: Number of participants in the workshop.
