# Supplementary material for: Co-Production of Behavior Change Intervention Promoting an Anti-Inflammatory Diet for Frailty Prevention in Community-Dwelling Older Adults
Source: Nutrients. 2026 Apr 30;18(9):1420. doi: 10.3390/nu18091420 (PMC13164891; doi:10.3390/nu18091420)
Supplement: Supplementary file 1 [file nutrients-18-01420-s001.zip › nutrients-4264391-supplementary.pdf]

Co-Production of Behavior Change Intervention Promoting an Anti-Inflammatory Diet for Frailty Prevention in Community-Dwelling Older Adults

Weida Lyu[1], Momoka Masuda[2], Kozue Kubo[1], Hayato Isomoto[1], Yuki Tamii[3], Youko Nakamae[3], Yuka Okitsu[3], Asako Arai[3], Masako Ueno[3], Masahiro Akishita[4], Katsuya Iijima[1, 5], Bo-Kyung Son[1, 5, 6*]

1 Institute of Gerontology, The University of Tokyo, Tokyo, Japan
2 Department of Human Ecology, The University of Tokyo, Tokyo, Japan
3 Welfare for the elderly in Toshima City, Tokyo, Japan
4 Tokyo Metropolitan Institute for Geriatrics and Gerontology, Tokyo, Japan
5 Institute for Future Initiatives, The University of Tokyo, Tokyo, Japan
6 Coproduction of Inclusion, Diversity and Equity (IncluDE) center, The University of Tokyo, Tokyo, Japan

* Correspondence: sontky72@g.ecc.u-tokyo.ac.jp

Table S1. Number of participants in the workshop

	Participants (n)		Participants of food habits survey (n)
	New	Total	
Session 1	13	13	18 (direct n=13, posting n=5)
Session 2	5	10	
Session 3	3	10	
Session 4	4	10	
Session 5	1	11	
Session 6	0	12	17 (direct n=12, posting n=5)
Total	25	66	15 comparable participants*

*15 comparable participants were engaged in WS averaged 4.0 $\pm$ 1.0 sessions across 6 sessions.
